# Making fair comparisons in pregnancy medication safety studies: An overview of advanced methods for confounding control

**DOI:** 10.1002/pds.4336

**Published:** 2017-10-17

**Authors:** Mollie E. Wood, Kate L. Lapane, Marleen M.H.J. van Gelder, Dheeraj Rai, Hedvig M.E. Nordeng

**Affiliations:** ^1^ PharmacoEpidemiology and Drug Safety Research Group, School of Pharmacy University of Oslo Norway; ^2^ Department of Quantitative Health Sciences University of Massachusetts Medical School Worcester MA USA; ^3^ Department for Health Evidence, Radboud Institute for Health Sciences Radboud University Medical Center Nijmegen The Netherlands; ^4^ Radboud REshape Innovation Center Radboud University Medical Center Nijmegen The Netherlands; ^5^ School of Social and Community Medicine University of Bristol UK; ^6^ Department of Child Mental and Physical Health Norwegian Institute of Public Health Oslo Norway

## Abstract

Understanding the safety of medication use during pregnancy relies on observational studies: However, confounding in observational studies poses a threat to the validity of estimates obtained from observational data. Newer methods, such as marginal structural models and propensity calibration, have emerged to deal with complex confounding problems, but these methods have seen limited uptake in the pregnancy medication literature. In this article, we provide an overview of newer advanced methods for confounding control and show how these methods are relevant for pregnancy medication safety studies.

## 1 INTRODUCTION

More than half of all pregnant women in Western countries take medication during pregnancy,^1^, ^2^, ^3^ making studies of medication safety a pressing public health concern. Studying medication safety in pregnancy presents particular challenges: Effects of medications on fetal development can be unpredictable, vulnerability to exposure changes during pregnancy, and outcomes may occur early in fetal development but be detected later.^4^ In the general population, knowledge of medication efficacy and safety is primarily based on randomized controlled trials. However, randomized trials routinely exclude pregnant women due to uncertainties about the effects of medications on fetal development, meaning that studies of medication safety in pregnancy must rely on reproductive toxicity studies in animals and on observational data in humans. Several landmark cases, such as the thalidomide disaster, have taught us that animal models for teratogenicity do not necessarily translate to humans. Observational studies, using data from cohort studies, registries, and administrative databases,^5^ are opportunities for understanding the risks of medication use in pregnancy, and in 2005, the Food and Drug Administration acknowledged that observational studies are the best method for assessing the maternal and fetal safety of using medication during pregnancy.^6^ However, confounding is a major source of bias in observational studies. Recent years have seen the rapid development of advanced methods for dealing with confounding, yet uptake of these methods has been slow in the pregnancy medication literature. This is unfortunate, because in this field, it is arguably especially important that researchers use the best methods for confounding control, because the consequences for getting the wrong answer are so profound: Failing to detect true effects of medication exposure can have enormous effects in the population, and falsely raising the alarm for a safe drug can result in women forgoing needed therapies and, in some cases, terminating wanted pregnancies.^6^

In this paper, we advocate for a greater use of advanced methods for confounding control in the pregnancy medication safety research field and provide an overview of these methods under the following framework:

1. How does this method help us to make fair comparisons between the exposed and unexposed groups?
2. How has this method been applied in the pregnancy medication literature?
3. How is the method used in practice?
4. What are the important assumptions for this method?
5. What are the major strengths and limitations of the method?

Table 1 provides an outline of pregnancy medication studies using advanced methods to deal with confounding. This paper gives a useful reference for both students and experienced researchers who wish to gain new skills in advanced methods for confounding control.

**KEY POINTS**

- Studies of the safety of medication use during pregnancy depend mainly on observational studies, which are subject to confounding bias.
- Novel methods for confounding control have seen limited uptake in the pregnancy medication safety literature.
- Application of novel methods is necessary to appropriately address the complex confounding scenarios found in pregnancy studies.

**Table 1:** Examples of application of advanced confounding control methods in the pregnancy medication safety literature

Medication and Study Reference	Outcome	Confounder(s)	Confounding Problem(s)			Method(s) Used				
			Time Varying	Complex/High Dimensional	Unmeasured Confounders	Propensity Scores/Summary Scores	Marginal Structural Models	Propensity Calibration	Sibling/Family Studies	Instrumental Variables
Ondansetron (Pasternak et al^14^)	Malformations	Nausea/vomiting; maternal characteristics, comorbidities, other medications, pregnancy history.		x		x				
Lithium (Patorno et al^15^)	Cardiac malformations	Maternal comorbidities, other medications, maternal characteristics.		x		x				
Statins (Bateman et al^17^)	Malformations	Maternal characteristics, obstetric and medical conditions, other medications.		x		x				
Triptans (Wood et al^25^)	Neurodevelopment	Other medications (time varying), maternal characteristics; migraine severity.	x				x			
Iron supplementation (Bodnar et al^26^)	Anemia	Maternal baseline characteristics; gastric symptoms; serum erritin and hemoglobin concentration.	x				x			
Triptans (Wood et al^35^)	Neurodevelopment	Other medications, maternal characteristics; migraine severity, attitudes about medication use.		x	x	x		x		
SSRI (Nezvalová‐Henriksen et al^41^; Viktorin et al^42^)	Gestational age, birth weight	Family factors, maternal depression; illnesses, other medications.		x		x			x	
Anti‐epileptic drugs (Bech et al^58^)	Spontaneous abortion	Severity of maternal epilepsy; maternal characteristics, environmental exposures, comorbidities.			x				x	
SSRI (Swanson et al^46^)	Maternal depression relapse	Maternal depression severity; comorbidities, other medications, maternal characteristics, proxies for severity.		x	x	x				x

Abbreviation: SSRI, selective serotonin reuptake inhibitor.

## 2 CONFOUNDING IN PREGNANCY MEDICATION STUDIES

**Figure 1:**
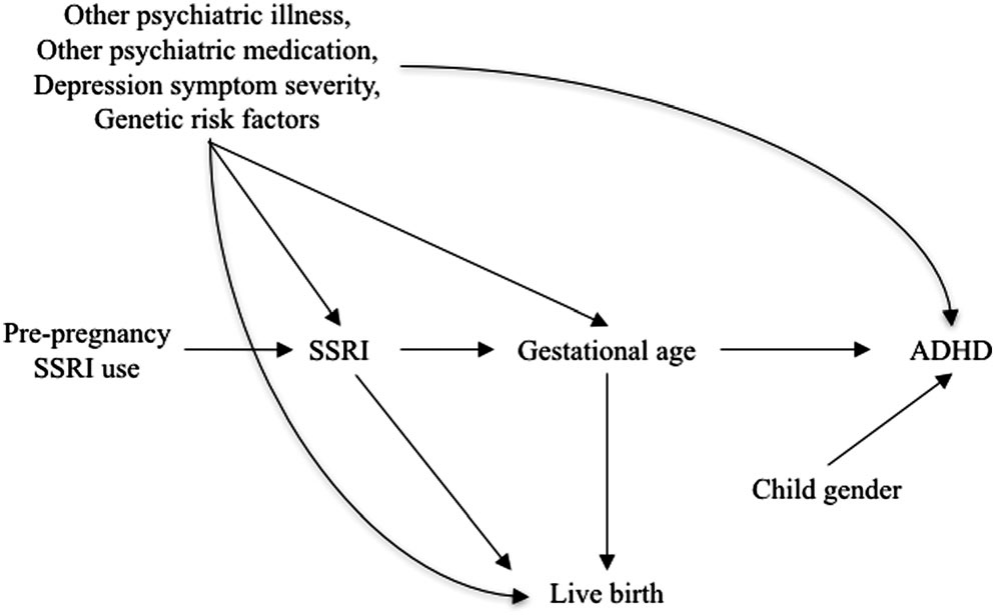
Conceptual model for the effect of prenatal selective serotonin reuptake inhibitor (SSRI) exposure on attention deficit/hyperactivity disorder (ADHD), including a set of important confounders (depression severity, concomitant medication use, and genetics), a potential mediator (gestational age), a collider (live birth), and factors related only to the exposure (prepregnancy SSRI use) or the outcome (child gender)

Confounding control begins with a review of the literature and consultation with subject‐area experts. Directed acyclic graphs (DAGs) provide a graphical means to represent the causal structure the investigator believes is present^7^ and guide study design, data collection, and analysis. Figure 1 is an example DAG showing one possible causal model for prenatal antidepressant exposure and childhood neurodevelopment, with potential biasing paths, including confounders (other psychiatric illness, other psychiatric medication use, depression severity, and genetics), which should be controlled as far as possible, as well as a mediator (gestational age), and a collider (live birth). Several nonbiasing paths, including a risk factor for the outcome that is unrelated to the exposure (child gender) and a predictor of exposure that is unrelated to the outcome (prepregnancy antidepressant use), are also shown. Obtaining unbiased effect estimates requires investigators to identify and control confounding, while avoiding bias from inappropriate control for colliders and mediators and loss of precision or confusing interpretation of estimates arising from control for factors only related to the exposure or outcome but not both.^8^ The Supporting Information contains a more comprehensive review of definitions of confounding, counterfactuals, and causal inference.

### 2.1 Methods for measured confounders

In Box 1 (Supporting Information), we include a simplified illustration of confounding by measured factors and the methods to address confounding.

Confounder summary scores and marginal structural models (MSMs) work by reducing a large amount of information about an individual into a single summary score. Two individuals can have the same summary score but different individual confounder values (eg, a woman with a propensity score [PS] for antidepressant use of 0.5 might be an unemployed smoker with anxiety, or a nonsmoking lawyer with depression), but because their distribution of confounders is equivalent, any differences in outcome will be attributable only to exposure to the drug of interest. Fair comparisons between exposure groups can then be made because within each stratum of exposure, the distribution of common causes of exposure and outcome is the same.

#### 2.1.1 Propensity scores (and other confounder summary scores)

The propensity score, which is the probability of exposure given observed confounders,^9^ reduces a large set of confounders to a single summary score. Propensity scores are commonly used in the medical literature; however, other summary score methods, including disease risk scores^10^ (preferred in the case of rare exposures) and polygenic risk scores^11^ (useful for cases when genetic confounding) are available.

Propensity scores are typically constructed using multivariable logistic regression, where exposure is the dependent variable and confounders are the independent variables. The PS model should include variables that are confounders or predictors of the outcome; inclusion of factors that are only predictors of exposure will increase variance without decreasing bias.^12^ High‐dimensional PSs, which include thousands of variables identified through computational algorithms, may also be useful for adjusting for unmeasured confounders, if the measured variables are partial proxies for the unmeasured confounders.^13^ The PS can be used to match, stratify, adjust, or weight the outcome model. Propensity scores, including high‐dimensional PS, have seen increased uptake in the pregnancy literature, ie, safety studies on ondansetron,^14^ lithium,^15^ antidepressants,^16^ and statins^17^ in pregnancy, but their use is still minimal compared to multivariable regression (Table 1). Box 1, in the Supporting Information, gives a simplified explanation of PS matching and weighting.

##### Assumptions

Use of PS requires several assumptions, including exchangeability (no unmeasured confounding) and positivity (nonzero probability of treatment). Neither assumption is formally testable. Positivity can be addressed by ensuring that the women in the sample all have the indication for the medication (ie, if assessing safety of antidepressants, all women in the sample should be at risk for treatment) and that no individuals with clear contraindications are included. Exchangeability is never assured; however, sensitivity analyses can yield estimates for how vulnerable an effect estimate may be to unmeasured confounding.

##### Strengths and limitations

PS is especially useful when working with a common treatment and rare outcome. They also separate the design of the study (modeling confounding) from modeling the outcome.^18^ However, for rare exposures, summary scores do not perform particularly well.^19^ In addition, use of PS methods may produce the appearance of effect modification and/or result in residual confounding in case control or case cohort studies^20^ or in cohort studies where exposure is misclassified.^21^

#### 2.1.2 Marginal structural models

Marginal structural models address time‐varying exposure and confounding.^22^, ^23^ Rules for confounder adjustment state we must adjust for common causes of the exposure and outcome, but should not adjust for factors on the causal pathway. In the case of time‐varying exposure and confounding, we encounter a double bind: Factors that are confounders in one part of the causal structure are mediators in another part (Figure S1A). For example, when studying the safety of antidepressants, we may wish to control for depression severity. However, antidepressant use in earlier pregnancy predicts depressive symptoms in later pregnancy, which will also predict subsequent antidepressant use. Standard adjustments for depression severity will always be biased in this scenario.

Central to the MSM is the inverse probability of treatment weight. At each measurement time *t*, the investigator uses logistic regression to construct the numerator (probability of exposure) and denominator (probability of exposure, given baseline predictors and history of exposure at time *t* − 1).^24^ The total weight is the product of the weights at each time point, and analyses are conducted in the weighted population, or *pseudo‐population*, in which individuals who are likely to be exposed are downweighted, while those who are unlikely to be exposed are upweighted, producing balance of measured confounders within strata of exposure.

Use of MSMs for pregnancy medication safety studies remains rare,^25^, ^26^ despite examples where timing of exposure is of great importance, and exposure is conditional on time‐varying confounders, such as other medication use, or changes in disease severity.

##### Assumptions

Under assumptions of positivity, exchangeability, and consistency, the MSM will give an unbiased estimate of the effect of the exposure on the outcome. These assumptions are not formally testable, although assessment of the positivity assumption may include evaluation of the inverse probability of treatment weight for extreme weights and progressive truncation of the weights to determine whether extreme weights are highly influential.^27^ When important confounders are unmeasured or incompletely measured, MSM methods will not provide unbiased effect estimates.

##### Strengths and limitations

The key strength of the MSM is that it allows consideration of time‐varying exposure and confounding, which is highly relevant in pregnancy research due to the changes in fetal vulnerability through the course of pregnancy and the tendency of women to change their medication use during pregnancy.^28^, ^29^ However, when the treatment‐covariate association is very strong, MSMs can produce very wide confidence intervals, which fail to include the true effect.^27^

### 2.2 Methods for incomplete confounder data

Failure to adjust for unmeasured confounders results in biased effect estimates (Figure S1B). In some situations, the confounder of interest was not measured in the original dataset, but was measured in a similar sample. In this scenario, confounder adjustment is possible, even if the outcome has not been measured in this sample, using PS calibration.^30^, ^31^, ^32^ Propensity score calibration is a method based on regression calibration^33^ that offers an additional advantage over other methods of calibration,^34^ by allowing for adjustment for multiple confounders. For example, in a study of triptan safety, we used a cross‐sectional study to jointly adjust estimates for migraine severity and type.^35^

In this method, 2 PSs must be calculated: the error‐prone PS (estimated in both the main and validation studies, including only the confounders available in the main study) and the gold‐standard PS (estimated in the validation study, including all confounders). The outcome model is fitted using the difference between the error‐prone and gold‐standard PSs to calibrate effect estimates.

##### Assumptions

In addition to the assumptions of PS models, outlined previously, PS calibration also assumes that the validation sample is a reasonable stand‐in for the main sample and that the measurement error model is correctly specified.^30^, ^31^ Propensity score calibration also assumes *surrogacy*, meaning that the error‐prone PS is an adequate surrogate for the gold‐standard PS.^36^ If the outcome is not measured in the validation study, the surrogacy assumption is not testable. Violations of surrogacy occur when the direction of confounding differs between the main and validation studies,^30^ and bias arising from violations of surrogacy can be predicted.^36^

Other methods exist for unmeasured confounding, including weighting by the inverse probability of missingness, as well as standard imputation techniques, and a comparison of these methods with PS calibration showed little material difference in bias reduction.^37^

##### Strengths and limitations

The main strength of PS calibration allows for adjustment for multiple unmeasured confounders. However, calibration methods fail when unmeasured confounding is strong, and violations of the surrogacy assumption may result in increased bias.

### 2.3 Methods for unmeasured confounding

Information on confounders may be too difficult to measure (eg, family environment or parenting style) or too costly (eg, deep sequencing genetic data). The methods discussed below exploit aspects of observational data to control for measured and unmeasured confounders.

#### 2.3.1 Sibling comparison designs

If the unmeasured confounders are shared between siblings (see Figure S1C for illustration), then studies examining with discordant exposure allows researchers to remove bias from shared confounders.^38^, ^39^, ^40^ If, for example, we believe that any differences in autism risk between children with and without prenatal exposure to antidepressants is due to inherited genetic risk, then comparing the autism diagnosis between pairs of siblings with different prenatal exposure should be less biased than comparing autism risk between unrelated exposed and unexposed groups.

There has been substantial uptake of sibling study designs in the pregnancy medication safety literature in recent years, particularly in studies examining the safety of antidepressants, where the main concern is separating the underlying genetic and familial components of depression from exposure to antidepressant medications.^41^, ^42^

##### Assumptions

Use of sibling designs is most appropriate when confounders that are shared between siblings are more important than unshared,^39^ and there are no carryover effects between siblings.^43^

##### Strengths and limitations

Sibling designs control measured and unmeasured confounding that is shared between siblings. However, failing to control for unshared confounders increases bias; sibling studies are also more vulnerable to bias from measurement error than nonsibling studies.^39^

#### 2.3.2 Instrumental variables

Instrumental variable (IV) methods^44^, ^45^ require identifying a variable whose effect on the outcome occurs only through the exposure: An example of a perfect instrument is a coin toss assigning an individual to exposure or nonexposure, while commonly used instruments include provider prescription preference and calendar time. One example is a study of antidepressant efficacy during pregnancy using provider preference, calendar time as a function of Food and Drug Administration recommendations, and geographic differences in antidepressant use as instruments; however, these instruments were only weakly associated with the treatment, which may have contributed to the equivocal findings.^46^ Instrumental variable studies are often conducted using a 2‐stage least squares methods, where in the first stage, the instruments are used as explanatory variables in a model predicting the exposure, and the predicted values from this first stage are used as predictors in the outcome model. Identifying a strong instrument that meets all assumptions is challenging, which has contributed to the slower adoption of this method. Mendelian randomization, which uses a genetic marker as an instrument, is a subtype of IV analysis^47^; while Mendelian randomization has not yet been used in pregnancy medication studies, studies estimating the effect of alcohol use during pregnancy on later neurocognitive outcomes have used the genetic variants encoding alcohol dehydrogenase, an enzyme that metabolizes alcohol, with some success.^48^

##### Assumptions

Instrumental variable analyses allow for unbiased effect estimation under strict assumptions: (1) The instrument has a causal effect on the exposure of interest, (2) the instrument effects the outcome only through the exposure, not through any other pathways, and (3) there are no common causes or confounders of the instrument‐outcome pathway (Figure S1D).

##### Strengths and limitations

Instrumental variable analyses control measured and unmeasured confounding, and so instruments that meet all the assumptions will mimic the results from a randomized trial. However, estimates are highly sensitive to violations of untestable assumptions, and violations may produce bias amplification.^44^

**Figure 2:**
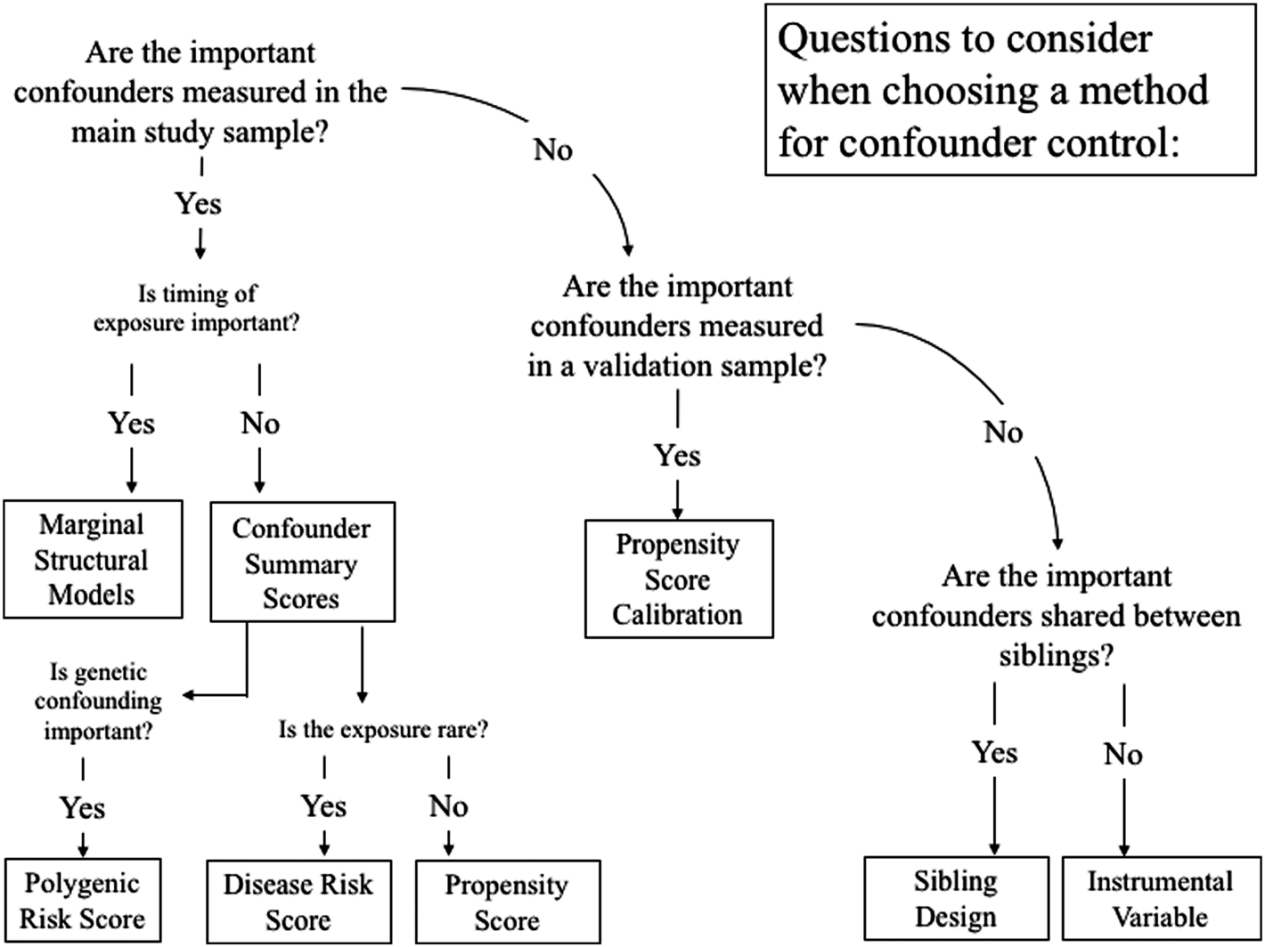
Choosing methods for confounding control

Figure 2 guides readers through selecting a method or methods, based on characteristics of confounder data. The most important first step is to draw a DAG or DAGs that represent the proposed causal mechanism, without regard to availability of data on confounders: If a confounder is important, it should be included in the DAG, even if the study did not collect data on it. Next, determine which confounders are available in your study and whether the data support the analytic method. For example, if your DAG shows that medication use and confounders vary over time, but your data shows no such variation, an MSM approach should not be used; if the data cannot identify siblings, this method cannot be used. Most importantly, we urge researchers to consider potential sources of confounding regardless of whether they were measured in the data and to choose the methods most suited to the data they have available: Figure 2 suggests a systematic way of approaching this process.

A reference to selected software for the methods discussed in this paper is included as part of the Supporting Information.

## 3 DISCUSSION

Studies of medication use during pregnancy use observational data to answer critical questions of safety and efficacy. More traditional methods for confounding control, such as stratification, restriction, matching, and adjustment have been described in great detail elsewhere, and because of this, we have not discussed them here. These older methods have their place in observational research, but as our understanding of the complexities of bias has progressed, so has our understanding of the limitations of these methods. The methods described in this paper were developed to address specific confounding problems and are necessary to reduce bias and, ultimately, to produce the best information possible to health care providers and pregnant women. Using these methods can produce substantially different results from traditional methods, such as when we compare the cohort and sibling studies of antidepressant safety,^41^, ^42^, ^49^ the regression‐adjusted estimates to the MSM estimates. Using multiple methods can also help to researchers triangulate, and it is reassuring when multiple methods, e.g. standard regression, PS methods, sibling controls, and negative paternal controls all produce similar estimates.^50^

With few exceptions, these methods have seen slow uptake in the pregnancy medication literature. This may be due to a sense of caution about methods that can seem opaque upon first encounter with the methods paper describing the technique. Caution is necessary when applying novel methods. However, it is also true that the standard regression methods require similar assumptions to the methods discussed in this paper. If readers find that their research question fits well with one of the scenarios described in this paper, we suggest approaching the problem by tackling the citations given for the technique. The techniques we describe in this paper have their roots in standard regression techniques and can be implemented with standard software.

While this paper focuses on bias due to confounding, other sources of bias such as exposure and/or outcome misclassification^51^ and selection bias,^52^ as well as seasonal effects,^53^ can also distort associations. This paper is not intended to be an exhaustive discussion of all possible methods for confounding control. New techniques are being developed all the time, and many of these, such as g‐estimation^54^, ^55^ and targeted maximum likelihood estimation,^56^ have not yet been implemented in the pregnancy medication literature. Quantitative bias analysis can help researchers account for bias from systematic errors in their data.^57^Further, the methods discussed herein are not mutually exclusive and can be used in combination with each other: Combining PSs with IVs^46^ or MSMs with quantitative bias analysis^25^ gives more information about the probable range of effect estimates than any single method.

Observational studies are vital to our understanding of medication safety in pregnancy, but great care must be taken in the analysis and interpretation of data to minimize confounding and bias. In all pharmacoepidemiological studies, sources of bias should be acknowledged and discussed and preferably quantified by performing sensitivity analysis of estimates under an array of assumptions about possible bias directions and magnitudes.

## ETHICS STATEMENT

The authors state that no ethical approval was needed.

## ACKNOWLEDGEMENT

The authors would like to thank Professor Sonia Hernandez‐Diaz, who contributed to early discussions about this article.

## CONFLICT OF INTEREST

The authors declare no conflict of interest.

## Supporting information

Directed acyclic graphs (DAGs) for **(a)**Time varying confounding: time‐varying exposure *A*, outcome *Y*, baseline confounders *C* and time‐varying confounders *TVC* at times 0, 1, and 2; **(b)**Unmeasured confounding: exposure *A*, outcome *Y*, and measured *C* and unmeasured *U* confounders; **(c)**Sibling study design, for siblings (1 and 2), with exposure *A*, outcome *Y*, and confounders *C* of *AY*, and shared unmeasured factors which cause *C*, *A*, and *Y*; **(d)** Instrumental variable (IV) which affects the outcome *Y* only through the exposure *A* and therefor controls both measured confounders *C* and unmeasured confounders *U*.
