# Supplementary material for: Making fair comparisons in pregnancy medication safety studies: An overview of advanced methods for confounding control
Source: Pharmacoepidemiol Drug Saf. 2017 Oct 17;27(2):140–7. doi: 10.1002/pds.4336 (PMC6646901; doi:10.1002/pds.4336)

Supplemental Material

Part 1. Discussion of confounding.

*Confounders and Causal Inference*

First, a definition of confounding: a confounder is a factor in a study that (a) is associated with (and precedes) the medication exposure, (b) is a risk factor for the outcome, and (c) does not lie on the causal pathway between medication use and outcome. In randomized studies, we can be reasonably confident that confounders (both measured and unmeasured) are balanced between exposed and unexposed groups, whereas in observational studies, this is almost certainly not the case. If the distribution of confounders is different for the exposed and unexposed groups, estimates of effect may be biased. Taking confounding into account is essential in observational studies. Further, it is important for researchers to carefully consider the timing of the confounder relative to the exposure and outcome.

Second, we focus on the definition of causal effects arising from the *counterfactual:* that is, we would like to understand what the experience of women who took a medication during pregnancy would have been, had she not taken the medication. In this definition, confounding exists when women who did not take the medication of interest are not adequate stand-ins for the women who did. The counterfactual approach to confounding clarifies a concept that researchers understand intuitively: that we must strive to make fair comparisons between exposed and unexposed groups. For example, for researchers studying the effects of antidepressant exposure on congenital malformations, comparing a group of women using antidepressants who also have severe depression to a group of women with no antidepressant use and no history of depression would be an unfair comparison: the unexposed group is not an adequate stand-in for the experience of women using antidepressants.

*Confounding in pregnancy medication studies*

Specific types of confounders arise often enough that they are grouped together. C*onfounding by indication* challenges the researcher to distinguish whether the outcome of interest is caused by the drug under study or the disorder being treated. Including a disease comparison group (women with the same disease and ideally the same disease severity, but not treated with the drug), or comparing drug use across different indications, offers advantages over studies comparing exposed cases to healthy controls only. *Confounding by concomitant medication use* occurs when women use multiple medications, and it is the concomitant medication, not the index drug, that is responsible for the observed effects. Other potential confounders include maternal factors such as lifestyle, paternal characteristics, genetic risk, and complex familial factors like home environment. These confounders may be measured with varying degrees of completeness or quality, depending on the data source, study design, and difficulty of measurement.


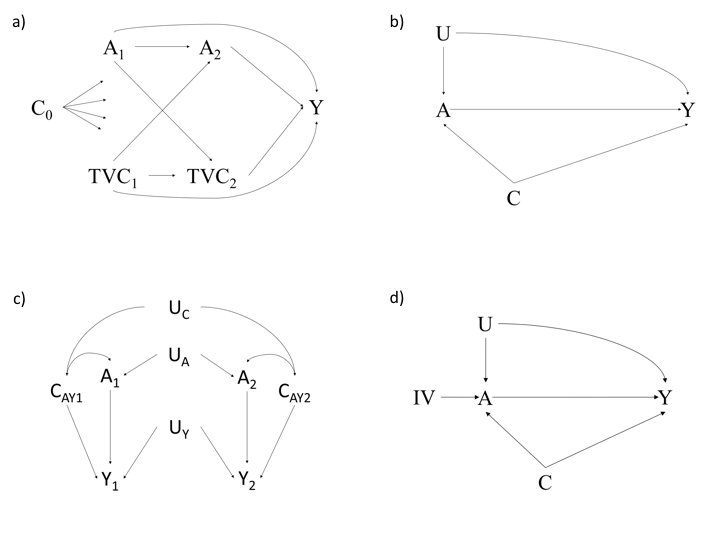


Figure S1. Directed acyclic graphs (DAGs) for **(a)** Time varying confounding: time-varying exposure *A*, outcome *Y*, baseline confounders *C* and time-varying confounders *TVC* at times 0, 1, and 2; **(b)** Unmeasured confounding: exposure *A*, outcome *Y*, and measured *C* and unmeasured *U* confounders; **(c)** Sibling study design, for siblings (1 and 2), with exposure *A*, outcome *Y*, and confounders *C* of *AY*, and shared unmeasured factors which cause *C*, *A*, and *Y*; **(d)** Instrumental variable (IV) which affects the outcome *Y* only through the exposure *A* and therefor controls both measured confounders *C* and unmeasured confounders *U*.


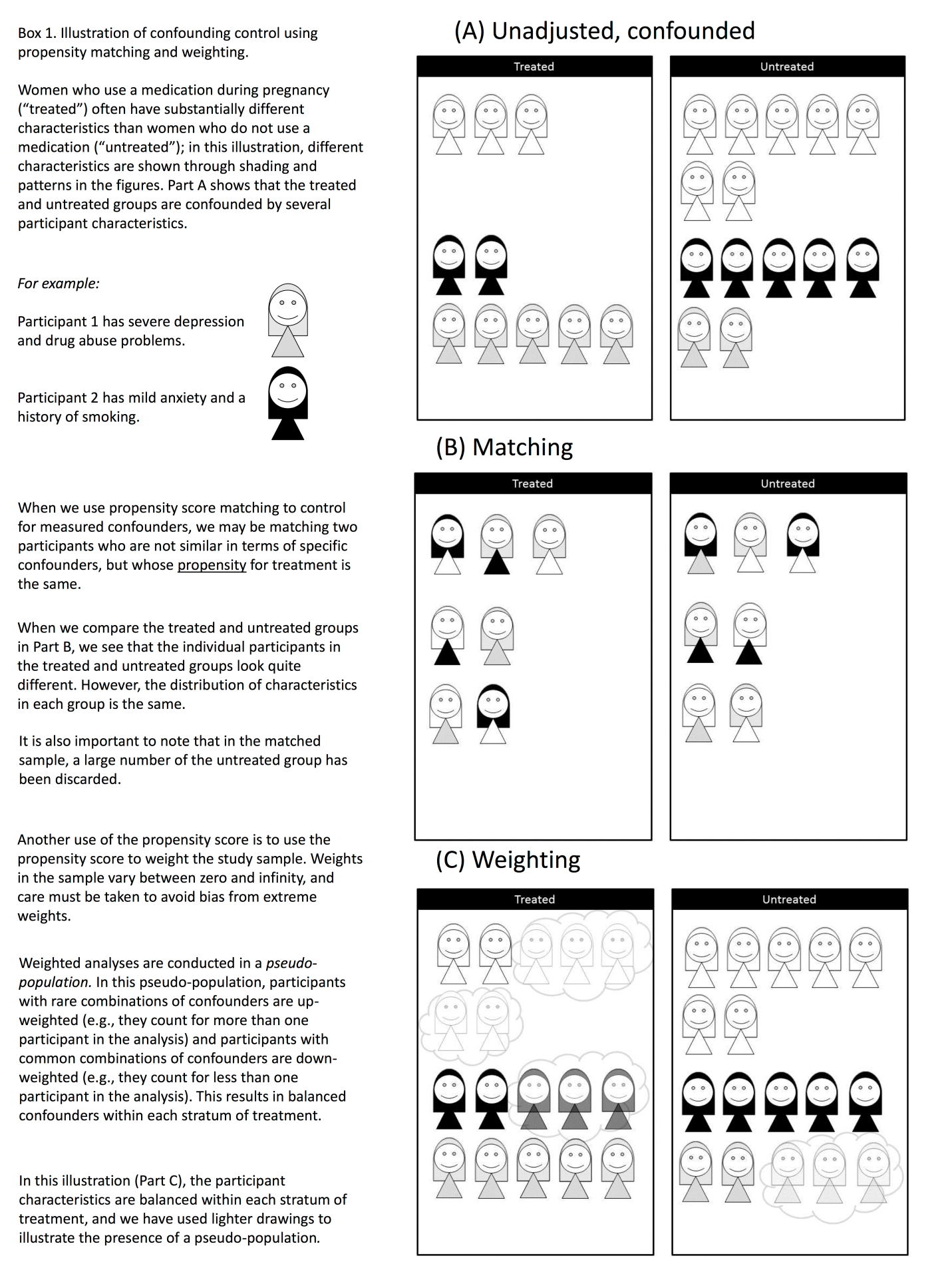

Supplement: Supplementary file 1 — Directed acyclic graphs (DAGs) for (a)Time varying confounding: time‐varying exposure A, outcome Y, baseline confounders C and time‐varying confounders TVC at times 0, 1, and 2; (b)Unmeasured confounding: exposure A, outcome Y, and measured C and unmeasured U confounders; (c)Sibling study design, for siblings (1 and 2), with exposure A, outcome Y, and confounders C of AY, and shared unmeasured factors which cause C, A, and Y; (d) Instrumental variable (IV) which affects the outcome Y only through the exposure A and therefor controls both measured confounders C and unmeasured confounders U. [file PDS-27-140-s001.docx]
